# Comprehensive Analysis of Sideroflexin 4 in Hepatocellular Carcinoma by Bioinformatics and Experiments

**DOI:** 10.7150/ijms.86990

**Published:** 2023-08-21

**Authors:** Zhipeng Du, Zhongchao Zhang, Xu Han, Huaping Xie, Wei Yan, Dean Tian, Mei Liu, Caijun Rao

**Affiliations:** 1 Department of Gastroenterology, Institute of Liver and Gastrointestinal Diseases, Tongji Hospital, Tongji Medical College, Huazhong University of Science and Technology, Wuhan, China.; 2 Department of Geriatrics, Tongji Hospital, Tongji Medical College, Huazhong University of Science and Technology, Wuhan, China.

**Keywords:** hepatocellular carcinoma, SFXN4, prognosis, proliferation, migration

## Abstract

**Background:** Sideroflexins (SFXNs) are a family of highly conserved mitochondrial transporters which regulate iron homeostasis and mitochondrial respiratory chain. However, the roles and mechanisms of SFXNs in HCC remain unknown.

**Methods:** SFXNs expression and prognostic value in HCC was comprehensively analyzed. Proteins interacting with SFXN4 were analyzed in STRING database. The co-expression genes of SFXN4 were analyzed in cBioPortal database, and function of SFXN4 co-expression genes were annotated. The putative transcription factors and miRNA targeting SFXN4 were analyzed in NetworkAnalyst. The correlation between SFXN4 expression and immune infiltration was analyzed by ssGSEA. Cancer pathway activity and drug sensitivity related to SFXN4 were explored in GSCALite. The roles of SFXN4 in proliferation, migration and invasion of HCC were assessed* in vitro* and *in vivo*.

**Results:** SFXN4 was consistently elevated in HCC, positively correlated with clinicopathological characteristics and predicted poor outcome. Functional enrichment showed SFXN4 was mainly related to oxidative phosphorylation, reactive oxygen species and metabolic pathways. SFXN4 expression was regulated by multiple transcription factors and miRNAs, and SFXN4 expression in HCC was associated with several cancer pathways and drug sensitivity. SFXN4 expression correlated with immune infiltration in HCC. *In vitro*, knockdown of SFXN4 inhibited HCC proliferation, migration and invasion, and decreased the expression of cyclin D1 and MMP2. *In vivo*, knockdown of SFXN4 inhibited the growth of tumor xenografts in mice.

**Conclusion:** SFXN4 was upregulated in HCC, predicted poor prognosis, and may facilitate HCC development and progression via various mechanisms. For HCC, SFXN4 may provide both prognostic information and therapeutic potential.

## Introduction

In 2020, hepatocellular carcinoma (HCC) occupies the sixth place in terms of cancer diagnoses, and the third place in terms of cancer-related deaths, which causes approximately 830,000 deaths worldwide, and the mortality is predicted to surpass 1,300,000 in 2040 1, 2. Despite improvement in therapeutic strategies, HCC patients continue to experience low long-term survival rates, owing to recurrence or metastasis after hepatic resection and non-responsiveness to targeted systemic therapies 3. Therefore, to develop novel therapeutic avenues, further investigation of the pathogenesis of HCC is necessary.

As the metabolic hub of cells, mitochondria can shape metabolic reprogramming and immune responses, which contribute significantly to development and progression of HCC 4, 5. Sideroflexins (SFXNs) are a family of evolutionarily conserved mitochondrial transporters in eukaryotes, which consist of 5 members (SFXN1-SFXN5). SFXNs are categorized under solute carrier 56 (SLC56), these transporters are located in the inner mitochondrial membrane, and contain 4 to 6 transmembrane domains 6, 7. As the founding member of SFXN family, SFXN1 was firstly reported in the flexed-tail (f) mouse with sideroblastic anemia, due to the fact that sideroblastic anemia is characterized by mitochondrial iron accumulation, SFXN1 was therefore deemed as a protein related to mitochondrial iron transportation and utilization 6. Recently, it has been discovered that SFXN1 plays a role in the mitochondrial iron transport process in several pathological states. In apelin-13-induced hypertrophic cardiomyocytes, SFXN1 was upregulated and transported the cytoplasmic iron (Fe^2+^) into mitochondria, led to mitochondria iron overload, attendant mitochondrial reactive oxygen species production, and the subsequent cardiac hypertrophy 8. Similarly, in sepsis-induced cardiac injury, acute lung injury and liver injury, SFXN1 transported cytoplasmic Fe^2+^ into mitochondria, generated mtROS, induced mitochondrial dysfunction, caused ferroptosis of cardiomyocytes, lung epitheliums and hepatocytes, and finally resulting in cardiac, lung, and liver injury 9-11. Importantly, SFXN1 functions as a mitochondrial serine transporter during one-carbon metabolism 12, and recent study revealed that serine active site containing 1 (SERAC1) was involved in SFXN1-mediated mitochondrial serine transport, while SERAC1 deficiency disrupted the one-carbon cycle and the balance of nucleotide pool, and caused depletion of mitochondrial DNA, suggesting that SFXN1 might be related to mitochondrial DNA generation 13. Apart from mitochondrial serine transport, Acoba et al. found that SFXN1 participated in the metabolism of coenzyme Q, heme, and α-ketoglutarate, and therefore supporting the function of complex Ⅲ in mitochondrial respiratory chain 14. SFXN1 overexpression enhanced proliferative, migratory and invasive capabilities, while inhibits apoptosis in lung adenocarcinoma cell 15. Up till now, reports about SFXN2 to SFXN5 were relatively limited. SFXN2 was reported to regulate mitochondrial iron homeostasis 16, which was elevated in multiple myeloma and promotes cell proliferation by impeding mitochondrial autophagy and enhancing iron-induced energy production 17. SFXN3 was enriched in neurons 18, which regulated synaptic morphology 19 and the neurodegeneration pathway 20, and SFXN3 overexpression was correlated with immunosuppressive microenvironment in head and neck squamous cell carcinoma and provided prognostic information 21. SFXN4 regulated iron metabolism, mitochondrial respiration, and heme metabolism 22, and SFXN4 deficiency caused macrocytic anemia and mitochondriopathy 23. Study on SFXN5 was rare, and the limited studies indicated that SFXN5 gene variation might be correlated with interstitial lung disease 24. It remains unknown, however, what the roles and mechanisms of SFXNs are in pathogenesis of HCC.

Here, we comprehensively analyzed SFXNs expression in HCC for the first time. Then, the predicted pathways, the regulatory mechanism, the correlation with immune infiltration of SFXN4 in HCC were investigated by bioinformatics methods. Furthermore, cellular experiments were performed to evaluate the impact of SFXN4 on HCC proliferation, migration and invasion, and the growth of tumor xenografts was assessed in mice. Our finding suggested that SFXN4 was upregulated in HCC, predicted poor prognosis, and may facilitate HCC development and progression via various mechanisms, indicating SFXN4 may provide both prognostic information and therapeutic potential for HCC.

## Materials and methods

### TCGA database analysis

The UCSC XENA-TCGA and Genotypic Tissue Expression (GTEx) (https://xenabrowser.net/datapages/) and The Cancer Genome Atlas (TCGA, https://portal.gdc.com) were used to determine SFXNs expression levels in pan-cancer and liver hepatocellular carcinoma (LIHC/HCC) cohort. The HCC cohort contains 371 cases with complete clinicopathological data. Since the clinical information for HCC cohort were acquired from online database, it is deemed that the written informed consent has been already obtained.

### GEPIA database analysis

An interactive website called Gene Expression Profiling Interactive Analysis (GEPIA) (http://gepia.cancer-pku.cn/) is available for analyzing RNA-seq data obtained from the TCGA database 25.

### GEO database analysis

An interactive open database called gene expression omnibus (GEO) (https://www.ncbi.nlm.nih.gov/geo/) contains array- and sequence-based data about gene expression profiles 26. SFXNs expression was explored in the six GEO datasets related to HCC, including GSE25097, GSE6764, GSE36376, GSE54236, GSE63898 and GSE76427.

### UALCAN database analysis

In addition to TCGA, an interactive web tool called UALCAN (http://ualcan.path.uab.edu/) also provides expression level of genes in 31 cancer types and the relevant clinical information 27. Here, SFXN4 expression in different tumor grade of HCC was analyzed in UALCAN. Furthermore, this website provides a Kaplan-Meier plot showing the prognostic value of SFXN4 for patients with HCC.

### STRING database analysis

A database called STRING (https://string-db.org/) contains information about known and putative interactions between proteins 28. The STRING database was explored to analyze the proteins interact with SFXN4.

### cBioportal database analysis

An interactive web called cBioPortal (http://www.cbioportal.org/) is utilized to analyze information about cancer genomics 29. In this study, we collected the top 50 co-expression genes of SFXN4, analyzed the interaction between SFXN members, and the correlation between SFXN4 and its putative interacting proteins obtained from the STRING database in cBioPortal.

### DAVID database analysis

A bioinformatics resource called DAVID (https://david.ncifcrf.gov/) is used to annotate gene function 30. To perform the functional annotation and enrichment analysis, we analyzed Gene Ontology (GO) and Kyoto Encyclopedia of Genes and Genomes (KEGG) of top 50 SFXN4 co-expression genes in DAVID. The GO analyses include biological processes (BP), cellular components (CC), and molecular functions (MF). According to the KEGG analysis, the putative pathway was mapped in relation to the top 50 SFXN4 co-expression genes.

### NetworkAnalyst database analysis

The NetworkAnalyst (https://www.networkanalyst.ca/) provides gene expression profiles and network visualizations 31, in which the regulatory networks of transcription factors and miRNA targeting SFXN4 were analyzed.

### ssGSEA analysis

In order to confirm whether SFXN4 expression correlated with 24 types of immune infiltration in HCC, we conducted single-sample gene set enrichment analysis (ssGSEA) utilizing the GSVA package in R (3.8.0) 32, and the correlation was analyzed by Spearman correlation.

### GSCALite database analysis

An online platform called GSCALite (http://bioinfo.life.hust.edu.cn/web/ GSCALite/) is utilized for analyses of cancer pathway activity and drug sensitivity 33.

### Cell culture

Human HCC cell line MHCC97H were provided by the Cell Bank of the Chinese Academy of Sciences (Shanghai, China), and Huh7 were obtained from the Japanese Cancer Research Bank (Tokyo, Japan), the two cell lines were stored in our institute and cultivated in a humidified atmosphere containing 5% CO2 at 37 ℃. The culture medium is Dulbecco's modified Eagle's medium (DMEM), which is supplemented with 10% heat-inactivated fetal bovine serum (Gibco, USA),100ug/ml penicillin and 100 μg/ml streptomycin.

### RNA extraction and quantative real-time PCR (qRT-PCR)

The RNA extraction and qRT-PCR were performed following the standard procedures as previously described 34. The primer sequences were presented as follows, cyclin D1, sense: 5'-GCTGCGAAGTGGAAACCATC-3', antisense: 5'-CCTCCTTCTGCACACATTTGAA-3'. matrix metalloproteinase 2 (MMP2), sense: 5'-CCAGATGTGGCCAACTACAA-3', antisense: 5'-GGTCAGGTGTGTAACCA ATGA-3'. GAPDH, sense: 5'-GCACCGTCAAGGCTGAGAAC-3', antisense: 5'-TGGTGAAGACGCCAGTGGA-3'.

### Western blot analysis

Forty-eight hours after siRNA transfection, RIPA lysis buffer (Servicebio Technology, China) containing Proteinase Inhibitor Cocktail (MedChemExpress, NJ, USA) was used to lyse Huh7 and MHCC97H cells. The BCA kit (Servicebio) was applied to quantify concentration of protein. After electrophoresis, 30 μg of total proteins were separated by SDS-PAGE, then, the proteins were transferred to PVDF membranes (Millipore, USA), and the membranes were immersed in 5% skimmed milk for blocking, and then incubated individually with specific primary antibodies [the internal control α-tubulin polyclonal antibody (Proteintech, China; 1:5000), SFXN4 antibody (Affinity, USA; 1:1000), cyclin D1 antibody (Proteintech, China; 1:2000), MMP2 (Boster, China; 1:200)]. After incubation at 4 ℃ overnight, the PVDF membranes were washed and then immersed in HRP-conjugated secondary antibody for 1 h. An ECL kit (NCMBIO, China) was applied for visualization of protein bands. The density of the bands was quantified by ImageJ software (NIH, USA).

### Transfection of siRNA or lentivirus

At a density of 2 × 10^5^ cells/well, the Huh7 and MHCC97H cells were plated in a 6-well plate. After 24 h, Lipofectamine 3000 (Invitrogen, USA) was used to transfect the cells with SFXN4 siRNAs or non-specific siRNAs in accordance with the manufacturer's instructions. After 5-7h, the cells were washed and cultivated in complete medium, the total RNA or protein were extracted 48 h or 72 h after transfection, and the transfection efficiencies were measured by Western blotting analysis. The siRNAs were purchased from Tsingke Biotechnology Co., Ltd (Beijing, China). The targeting sequences are as follows, siRNA-1: 5'-GCAGCGTTCAACAGCATCAAT-3' and siRNA-2: 5'-CGAGGCAACTATTGTGCACAA-3'. Based on the sequence of siRNA-1, the SFXN4-knockdown lentivirus (Lv-shSFXN4) was constructed by Tsingke Biotechnology Co., Ltd. MHCC97H cell were transfected with Lv-shSFXN4 and the relevant control lentivirus (Lv-shcontrol), and 72 h after transfection, the cells were subjected to DMEM supplemented with puromycin for 14 days, and stable SFXN4 knockdown cells were established, as previously described 34.

### Cell Counting Kit-8 (CCK-8) assay

The indicated Huh7 and MHCC97H cells were subjected to CCK-8 kit (Beyotime, China) to assess proliferation capability, as previously described 34. Twenty-four hours after siRNA transfection, a number of 1,000 indicated Huh7 and MHCC97H cells were seeded into 96-well plates and then cultivated. The culture media were taken from each well at each time point (24h, 48h, 72h, 96h), and PBS was used to wash the cells thrice. A mixture of CCK-8 (10 μl) and complete medium (100 μl) was added. Then, the plates were placed at 37°C, and incubated for another 2 h. The absorbance of cells was determined at 450 nm by a microplate reader (Biotek, USA).

### 5-ethynyl-2'-deoxyuridine (EdU) assay

Based on the previous description, we performed the EdU assay to further measure the proliferation capability 35. In brief, 24 h after siRNA transfection, 1× 10^5^ Huh7 and MHCC97H cells were plated in 24-well plates, and cultivated for another 24 h. Then, 10 µM EdU solution (Beyotime) was added to each well, and the plates were placed back to the incubator for 2 h incubation. In the dark, cell nuclei were then stained by DAPI (Beyotime) for 30 min. The EdU-positive cells were observed and analyzed by an Olympus fluorescence microscope.

### Transwell assay

Transwell^TM^ chambers (pore size, 8 microns; Corning) were applied to measure HCC migration and invasion capabilities. The chambers were placed inside 24-well cell culture plates, pre-coated with (invasion assay) Matrigel (BD Bioscience, USA) or not (migration assay). Twenty-four hours after siRNA transfection, the indicated cells (5 × 10^4^ for migration assay and 1 × 10^5^ for invasion assay) were re-suspended in 200 μl serum-free DMEM medium, and then seeded into upper chamber of inserts, respectively, with the lower chamber containing 600 µl of complete medium. Twenty-four hours after incubation, we scraped the upper surface of inserts to remove unmigrated cells. Crystal violet (Beyotime) was used for staining the cells that migrated across the membrane after 20 minutes of fixation with 4% paraformaldehyde. Using a microscope, we observed and analyzed the cell numbers in five random fields of view.

### Mouse xenograft tumor assay

The mouse experiment was approved by the Ethics Committee of Animal Experiments of Tongji Medical College, Huazhong University of Science and Technology. The BALB/c nude mice (male, 4 weeks old) were purchased from Huafukang Bioscience Ltd (Beijing, China), and bred in pathogen-free conditions.

The mice were randomly divided to two groups (n=5), designated as shcontrol group and shSFXN4 group, respectively. MHCC97H cells (5×10^6^) stably transfected with Lv-shSFXN4 or Lv-shcontrol were separately mixed with matrix gel in 1: 1 ratio, and implanted subcutaneously into the flank of mice. The length (L) and width (W) of tumors were measured every three days, and the tumor volume (V) was measured by the following equation: V = (L×W^2^)/2. Three weeks later, the mice were executed and the subcutaneous tumors were obtained.

### Statistical analysis

In the Prism 5.0 GraphPad Software (La Jolla, CA, USA), the Student's t-test was conducted to analyze the data (presented as mean ± standard deviation) among the different groups. Spearman rank correlation coefficients were utilized to determine correlation of two groups. The predictive value of SFXN4 expression in prognosis was assessed by Kaplan-Meier method. If *P* < 0.05, the statistical significance was determined.

## Results

### SFXN4 expression is elevated in HCC and its high expression predicts poor survival

**Figure 1:**
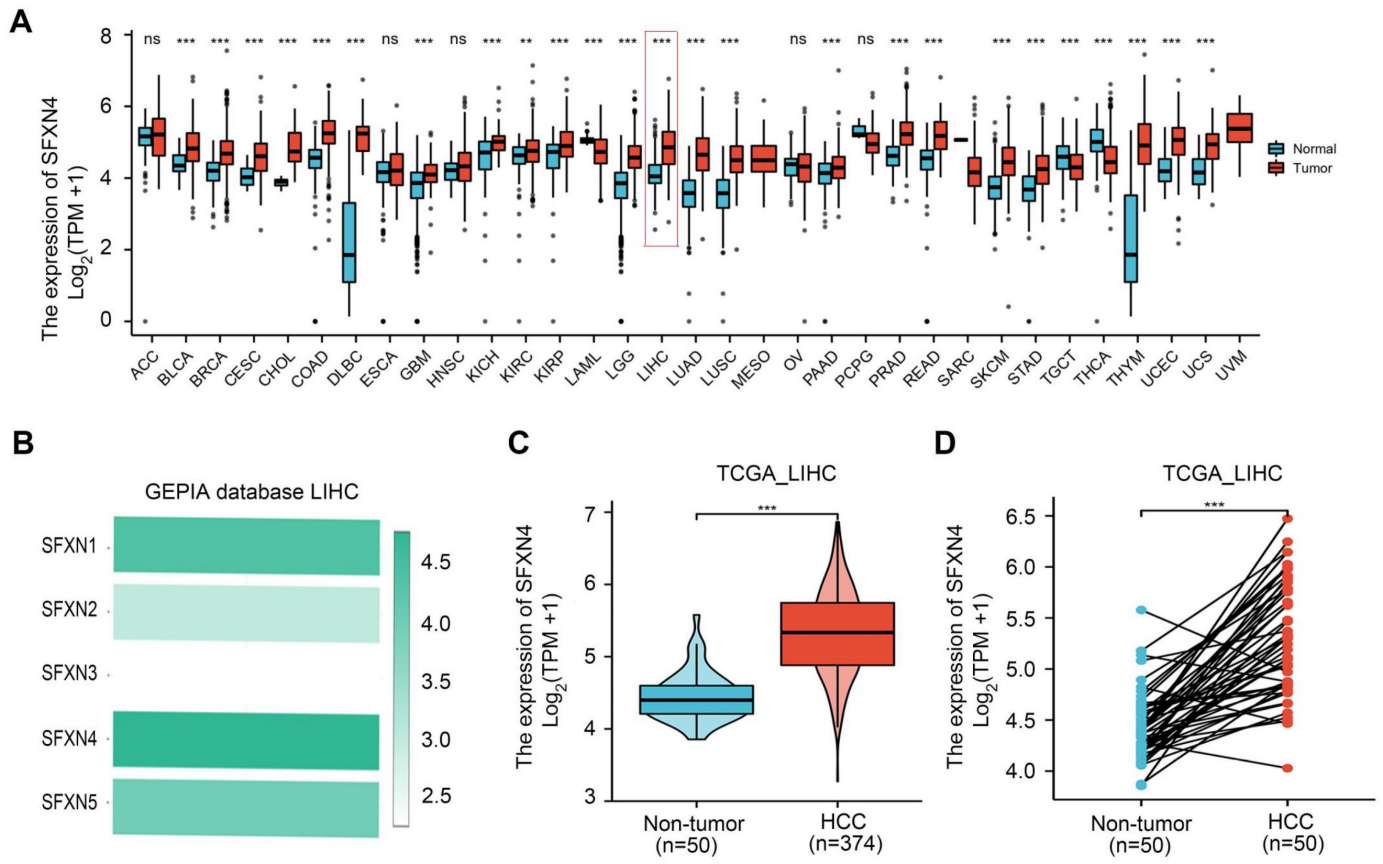
SFXN4 expression is elevated in HCC from TCGA database. (A) SFXN4 expression in pan-cancer from XENA-TCGA-GTEx database. (B) SFXN1-SFXN5 expression in HCC from GEPIA database. (C) SFXN4 expression in non-paired paracancerous non-tumor tissues and HCC tissues from TCGA database. Non-tumor, n=50; HCC, n=374. (D) SFXN4 expression in paired paracancerous non-tumor tissues and HCC tissues from TCGA database. Non-tumor, n=50; HCC, n=50. ^* *^P<0.01, ^***^P<0.001, ^ns^*P*>0.05.

To explore the expression pattern of SFXN family in HCC, we firstly analyzed SFXNs expression in pan-cancer data from TCGA and GTEx. Analysis of TCGA-GTEx database showed that, in normal and HCC, the expression levels of SFXN1 (4.543 ± 0.509 vs. 4.499 ± 0.826, *P* > 0.05) and SFXN2 (3.21 ± 0.58 vs. 3.123 ± 0.912, *P* > 0.05) were not dramatically changed, while SFXN3 (2.136 ± 0.805 vs. 2.394 ± 1.019, *P* < 0.01) and SFXN4 (4.15 ± 0.479 vs. 4.854 ± 0.636, *P* < 0.001) were dramatically upregulated in HCC samples, however, SFXN5 expression (4.5 ± 0.644 vs. 4.043 ± 0.739, *P* < 0.001) was decreased in HCC tissues (Figure 1A, Figure S1). Furthermore, the results from GEPIA database showed that, among the SFXN members, SFXN4 expression was the highest in HCC, while SFXN3 expression was the lowest (Figure 1B). In TCGA database, we found the expression of SFXN4 was higher in HCC tissues than in non-paired paracancerous non-tumor tissues (3.992 ± 0.576 vs. 3.247 ± 0.284, *P* < 0.001) (Figure 1C), and also compared to paired paracancerous non-tumor tissues (5.283 ± 0.556 vs 4.444 ± 0.335, *P* < 0.001) (Figure 1D).

**Figure 2:**
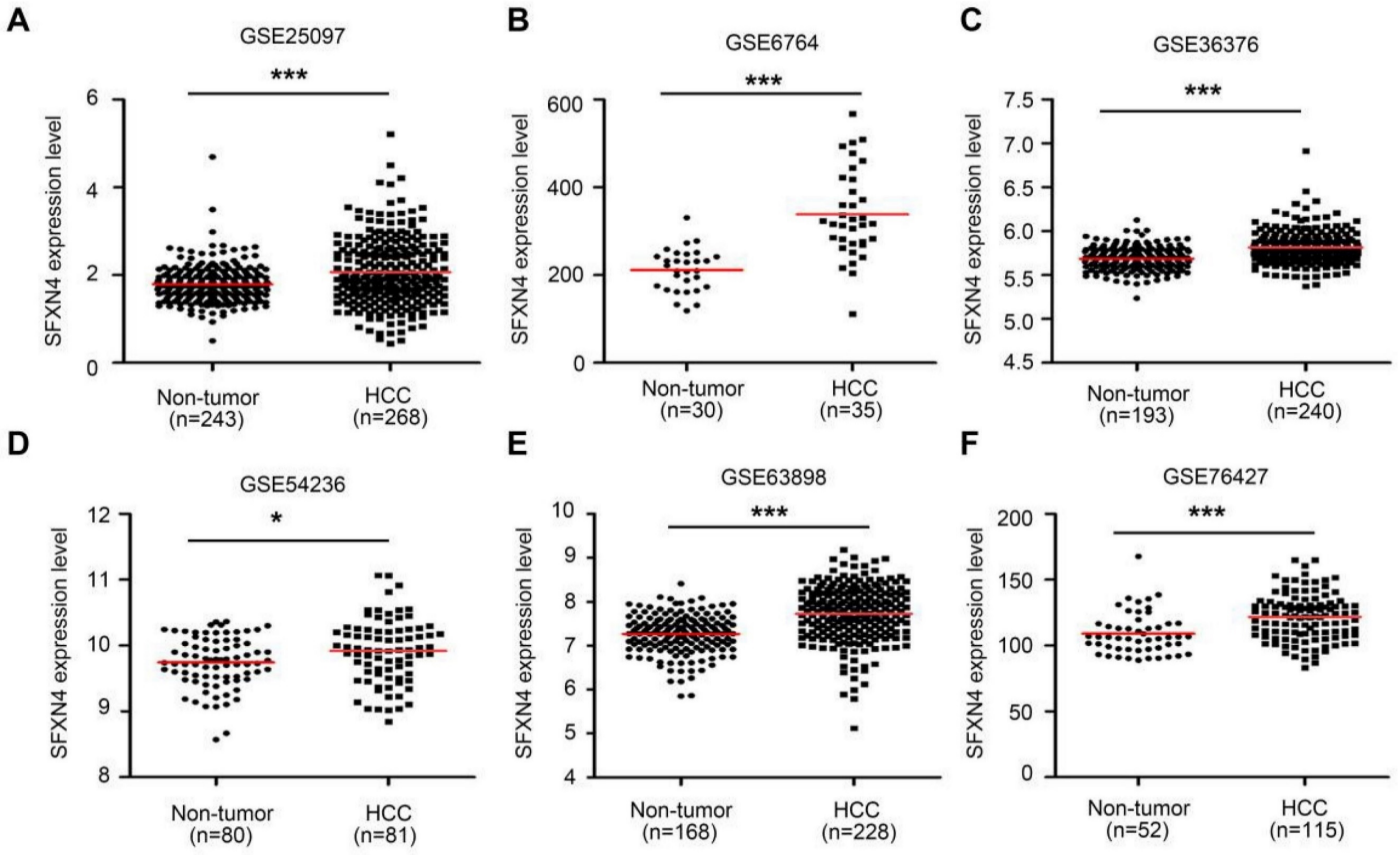
SFXN4 expression is elevated in HCC from GEO database. SFXN4 expression in unpaired paracancerous non-tumor tissues and HCC tissues from six GEO database. (A) GSE25097. (B) GSE6764. (C) GSE36376. (D) GSE54236. (E) GSE63898. (F) GSE76427. ^*^P<0.05, ^***^P<0.001.

Then, we further explored six GEO datasets to verify SFXNs expression in HCC. These data showed SFXN4 expression were consistently upregulated in all the six GEO datasets (Figure 2A-F); the expression of SFXN1 and SFXN5 in HCC tissues decreased in three datasets, while remained unchanged in the other three datasets; the expression of SFXN2 decreased in four datasets, increased in one dataset, and remained unchanged in one dataset; the expression of SFXN3 decreased in one dataset, and remained unchanged in five datasets (Table 1). Considering the expression consistency of SFXN members in different database and to guarantee the accuracy of this study, we selected SFXN4 as the target member for further study.

**Figure 3:**
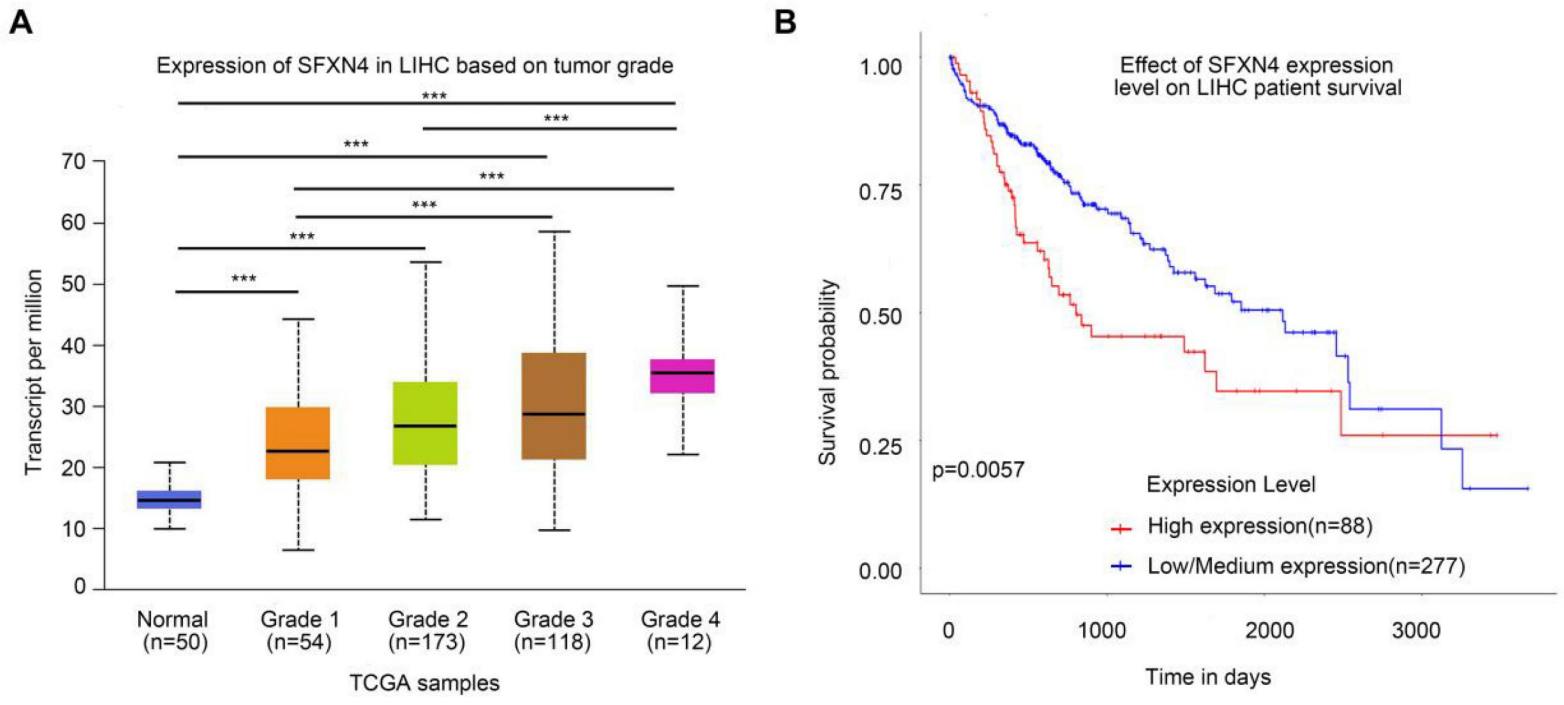
Correlation of SFXN4 expression with tumor grade and prognosis. (A) SFXN4 expression in different tumor grade of HCC from UALCAN. ^***^, *P*<0.001. (B) Correlation between SFXN4 expressions and overall survival of HCC patients from UALCAN. ^***^P<0.001.

To assess the prognostic value of SFXN4 expression for HCC patients, we first analyzed correlation of SFXN4 expression with clinicopathological factors in the TCGA database. The results demonstrated SFXN4 expression is correlated with race, weight, BMI, histologic grade, and the level of alpha-fetoprotein (AFP), which is a widely acknowledged biomarker for HCC (Table 2). Results from UALCAN database confirmed that SFXN4 expression gradually elevated along with the tumor grade proceeded (n = 407) (Figure 3A). Moreover, data from UALCAN database suggested, in HCC patients with high expression of SFXN4 (n = 88), survival was poorer than in patients with low/medium expression of SFXN4 (n = 277) (Figure 3B).

### GO and KEGG enrichment analysis of SFXN4 and its co-expression genes in HCC

**Figure 4:**
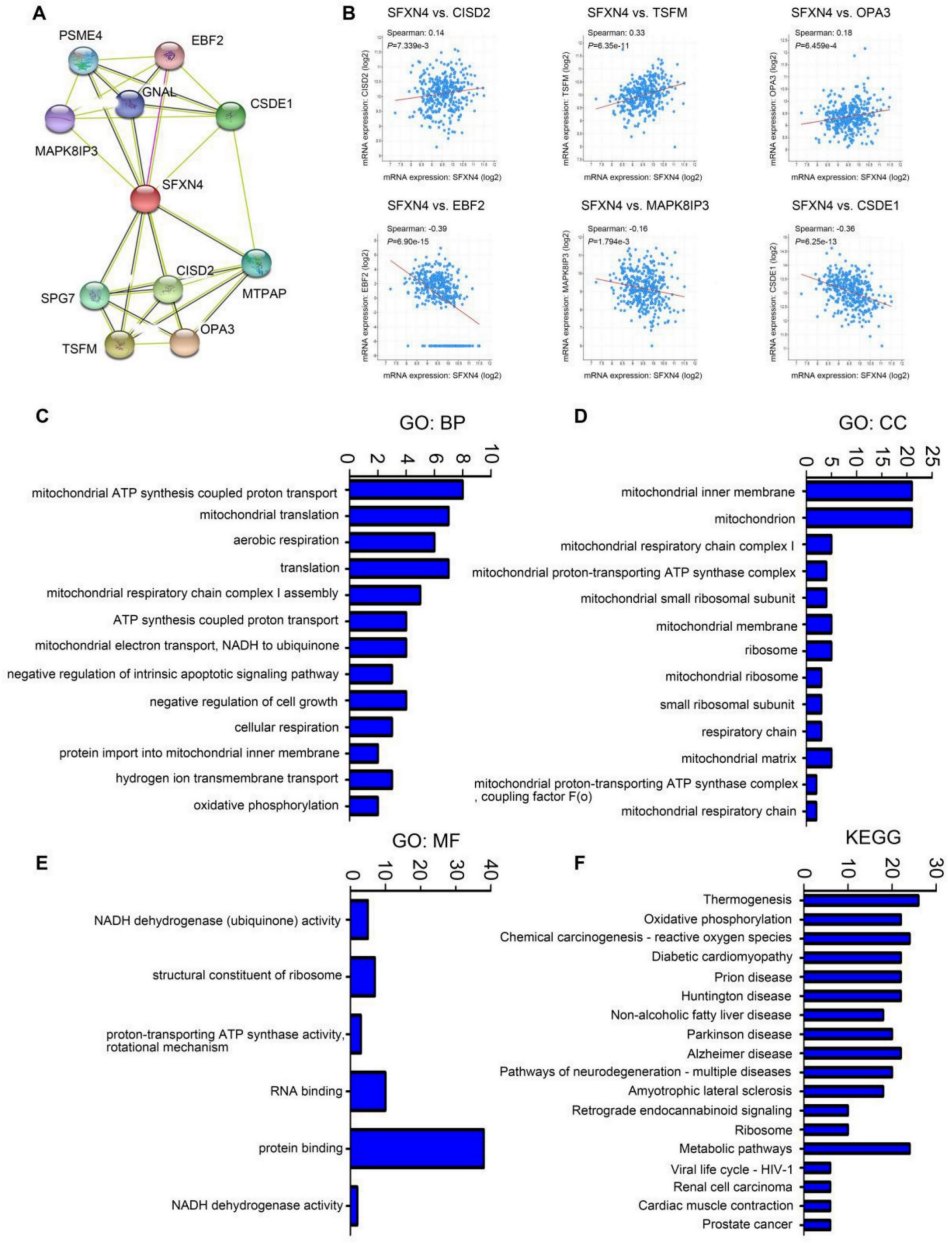
GO and KEGG enrichment analysis of SFXN4 and its co-expression genes in HCC. (A) PPI network (STRING). (B) Correlation between SFXN4 and its interacting proteins (cBioportal). (C-E) GO: BP, biological processes; CC, cellular components; MF, molecular functions (DAVID). (F) KEGG (DAVID).

Subsequently, we analyzed the GO and KEGG enrichment of co-expression genes associated with SFXN4. Firstly, we dissected the interacting neighbors of SFXN4 in STRING database, and found 10 potential SFXN4-related genes, including PSM4, EBF2, MAPK8IP3, GNAL, CSDE1, SPG7, CISD2, MTPAP, TSFM, OPA3 (Figure 4A). By analysis of these genes in cBioportal, it was determined that SFXN4 had positive correlation with CISD2 (p = 7.339e-3, which promoted HCC by regulating autophagy 36, TSFM (p = 6.35e-11, a mitochondrial elongation factor, associated with mitochondrial translation 37, and OPA3 (p = 6.459e-4, an outer mitochondrial membrane lipid metabolism regulator, which enhanced HCC cell proliferation and aerobic glycolysis 38, while SFXN4 was negatively related to EBF2 (p = 6.90e-15, an EBF transcription factor), MAPK8IP3 (p = 1.794e-3, mitogen-activated protein kinase 8 interacting protein 3), and CSDE1 (p = 6.25e-13, cold shock domain containing E1), and their roles in HCC were not reported yet (Figure 4B).

In addition, we collected the top 50 co-expression genes of SFXN4 in cBioportal database (Table 3), and analyzed the potential role of SFXN4 and its co-expression genes by GO and KEGG in DAVID database. We found that biological processes (BP), including GO:0042776 (mitochondrial ATP synthesis coupled proton transport), GO:0032543 (mitochondrial translation), GO:0009060 (aerobic respiration), GO:0032981 (mitochondrial respiratory chain complex I assembly), GO:0015986 (ATP synthesis coupled proton transport), GO:0006120 (mitochondrial electron transport, NADH to ubiquinone) were significantly regulated by SFXN4 in HCC (Figure 4C). In addition, SFXN4 regulated the cellular components (CC), such as GO:0005743 (mitochondrial inner membrane), GO:0005739 (mitochondrion), GO:0005747 (mitochondrial respiratory chain complex I), GO:0031966 (mitochondrial membrane) (Figure 4D). Furthermore, SFXN4 regulated the molecular functions (MF), especially GO:0005515 (protein binding), GO:0003723 (RNA binding) (Figure 4E). In the KEGG analysis, we found that SFXN4 was implicated in multiple diseases, and was mainly related to oxidative phosphorylation, reactive oxygen species and metabolic pathways (Figure 4F).

### Regulation analysis of SFXN4 in HCC

**Figure 5:**
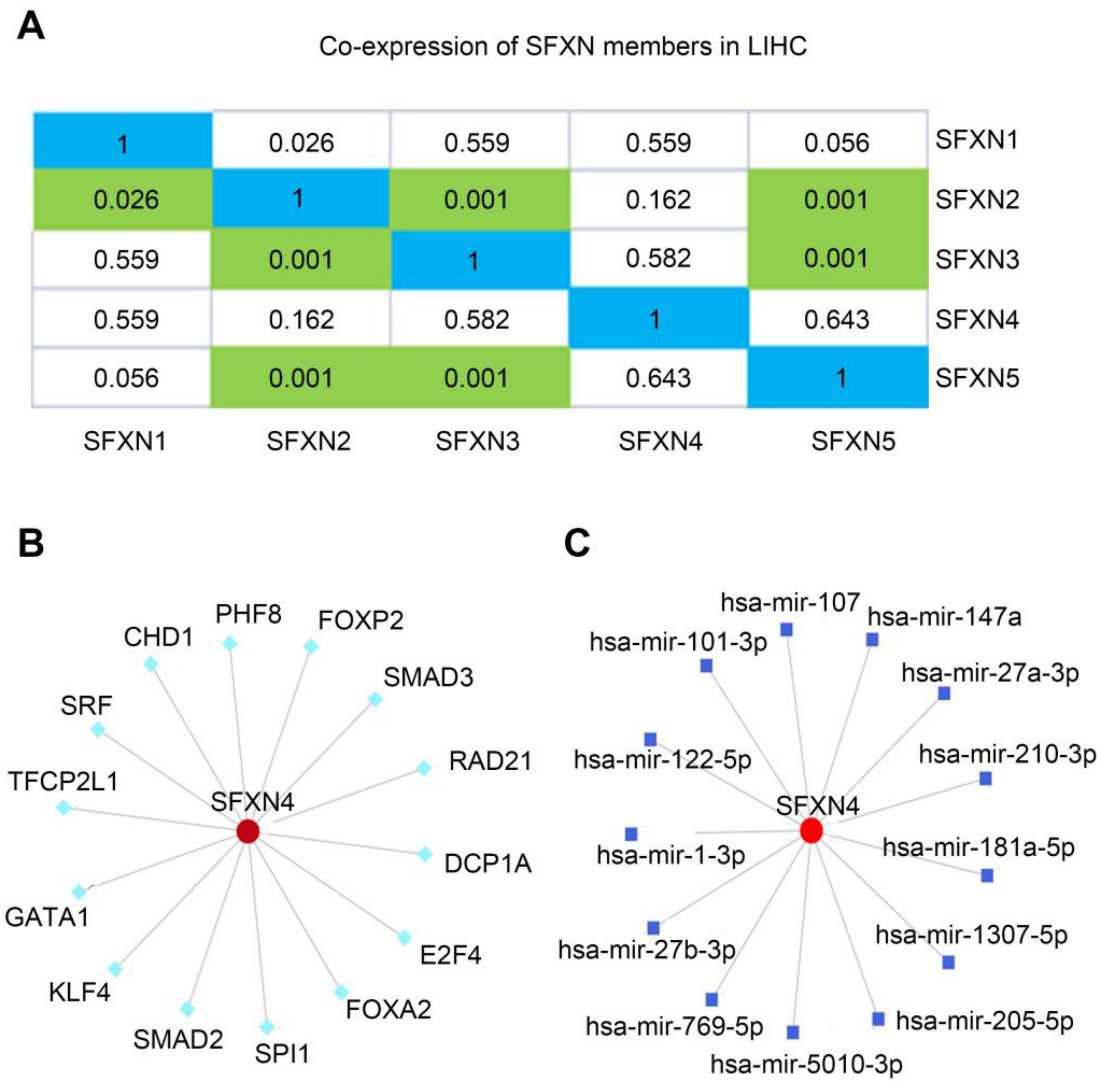
Regulation analysis of SFXN4 in HCC. (A) Correlation of different SFXN members with each other in HCC (cBioportal). (B) Transcription factors target SFXN4 (NetworkAnalyst). (C) miRNAs target SFXN4 (NetworkAnalyst).

Then, we explored the putative regulatory mechanism of SFXN4 expression. Firstly, we investigated whether SFXN4 mRNA expression was regulated by other SFXN members. Data in cBioportal showed that SFXN4 expression had no significant correlation with other SFXN members (Figure 5A). Then, transcription factors and miRNA targeting SFXN4 were analyzed in the NetworkAnalyst, which demonstrated SFXN4 was regulated by multiple transcription factors and miRNA, including Smad2, Smad3, FOXA2, FoxP2, hsa-mir-1-3p, hsa-mir-27-3p, etc. (Figure 5B-C).

### Correlation of SFXN4 expression with immune infiltration in HCC

**Figure 6:**
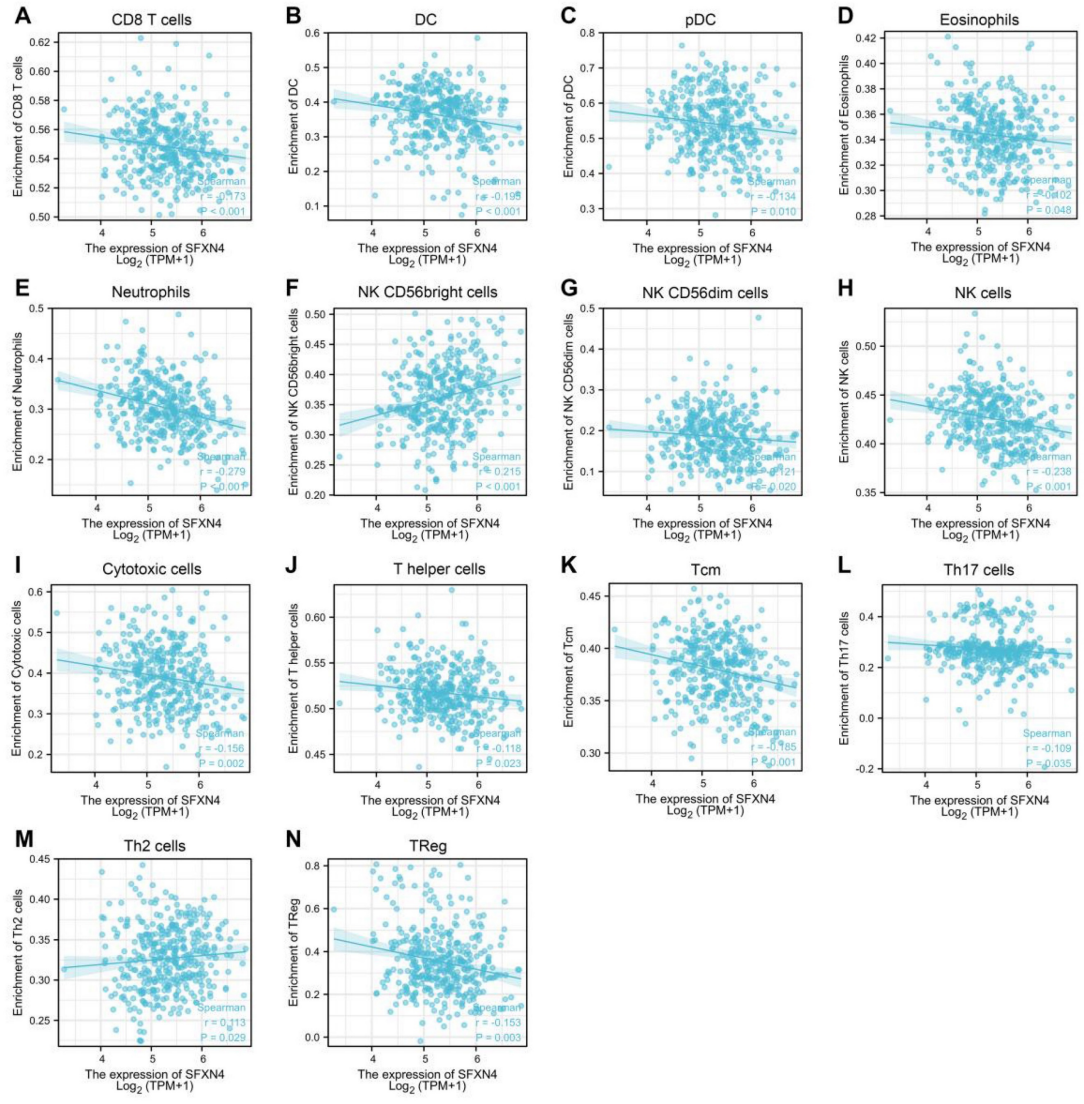
Correlation of SFXN4 expression with immune infiltration in HCC. (A) CD8 T. (B) DC. (C) pDC. (D) eosinophils. (E) neutrophils. (F) NK CD56bright cells. (G) NK CD56dim cells. (H) NK cells. (I) cytotoxic cells. (J) T helper cells. (K) Tcm. (L) Th17 cells. (M) Th2 cells. (N) TReg. The data were acquired from ssGSEA, and* P*<0.05 was considered statistically significant.

As immune infiltration exerts critical role in HCC progression 39, the correlation of SFXN4 with immune cell infiltration was analyzed in ssGSEA. The results showed SFXN4 expression correlated significantly with the levels of CD8 T (r = -0.173, *P* < 0.001), DC (r = -0.195, *P* < 0.001), pDC (r = -0.134, *P* = 0.01), eosinophils (r = -0.102, P = 0.048), neutrophils (r = -0.279, *P*<0.001), NK CD56bright cells (r = 0.215, *P* < 0.001), NK CD56dim cells (r = -0.121, *P* = 0.02), NK cells (r = -0.238, *P* < 0.001), cytotoxic cells (r = -0.156, *P* = 0.002), T helper cells (r = -0.118, *P*=0.023), Tcm (r = -0.185, *P* < 0.001), Th17 cells (r = -0.109, *P*=0.035), Th2 cells (r = 0.113, *P* = 0.029), TReg (r = -0.153, *P* = 0.003) (Figure 6A-N). Interestingly, we could find that the infiltration of most immune cells were negatively correlated with SFXN4 expression, except NK CD56bright cells and Th2 cells. However, SFXN4 expression did not correlate significantly with B cells, aDC, iDC, macrophages, mast cells, total T cells, Tem, TFH, Tgd, and Th1 cells (Figure S2A-J). Consistently, we speculated that these negative correlations supported the lack of association between SFXN4 with inflammation seen in Table 2. Accordingly, the results of these studies suggested that SFXN4 expression correlated with immune infiltration.

### Cancer pathway activity and drug sensitivity analysis of SFXN4 in HCC

**Figure 7:**
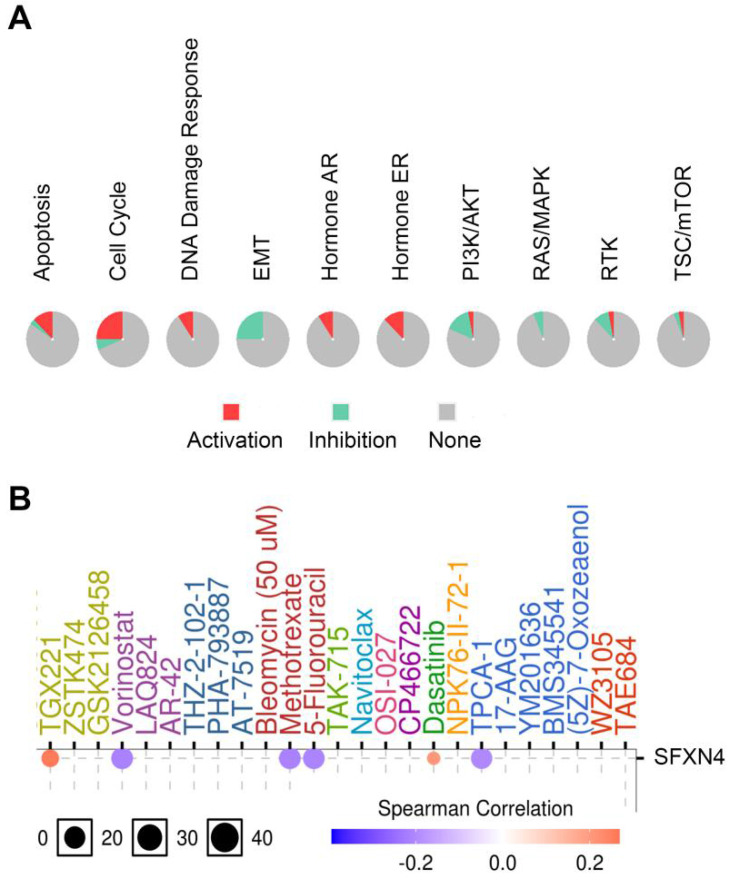
Cancer pathway activity and drug sensitivity analysis of SFXN4 in HCC. (A) Potential cancer pathway activity regulated by SFXN4 in HCC. The color red (or green) indicates the percentage of HCCs in which pathways may be activated (or inhibited) by SFXN4 (GSCALite). (B) Drug sensitivity analysis of SFXN4 in HCC. Positive (or negative) correlation indicated high expression of SFXN4 is resistant (or responsive) to the drug (GSCALite).

Considering that SFXN4 was closely related to HCC, we analyzed the potential pathway through which SFXN4 might exert it roles and the drug sensitivity in patients with HCC in GSCALite database. SFXN4 mainly activated apoptosis pathway, cell cycle pathway, DNA damage response, hormone AR and hormone ER, and SFXN4 mainly inhibited EMT pathway, PI3K/AKT pathway, RAS/MAPK, RTK (Figure 7A).

As for drug sensitivity analysis, SFXN4 was predicted to be responsive to Vorinostat (an inhibitor targeting HDAC 1, HDAC2, HDAC3, HDAC6, HDAC7, and HDAC11), Methotrexate (adihydrofolate reductase inhibitor), 5-Fluorouracil (athymidylate synthase inhibitor) and TPCA-1 (an inhibitor for IKK2), while resistant to TGX221 (a Keap1-Nrf2 inhibitor) and Dasatinib (a dual inhibitor for Src/Bcr-Abl), suggesting that SFXN4 might be a biomarker for drug screening (Figure 7B).

### SFXN4 knockdown inhibits HCC proliferation, migration and invasion *in vitro*, and inhibits HCC growth *in vivo*

**Figure 8:**
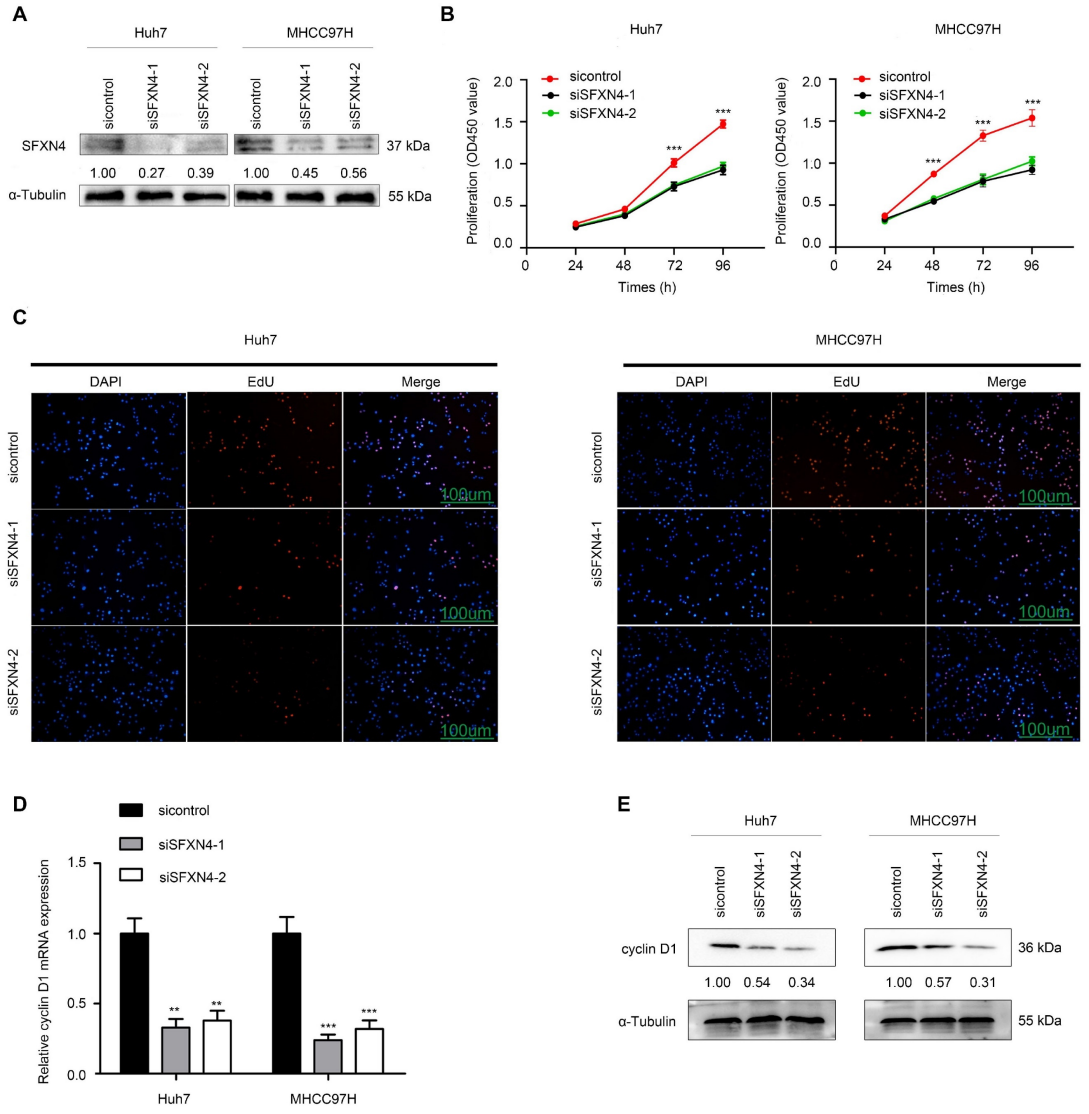
SFXN4 knockdown inhibits HCC proliferation. (A) Western blot was performed to assess siRNA transfection efficiency. (B-C) The CCK-8 (B) and EdU assays (C) were performed to measure Huh7 and MHCC97H proliferation. (D-E) The qRT-PCR and Western blot were performed to detect cyclin D1 mRNA (D) and protein (E) expression in indicated cells. ^***^P<0.001, ^**^P<0.01. Scale bars: 100 μm.

**Figure 9:**
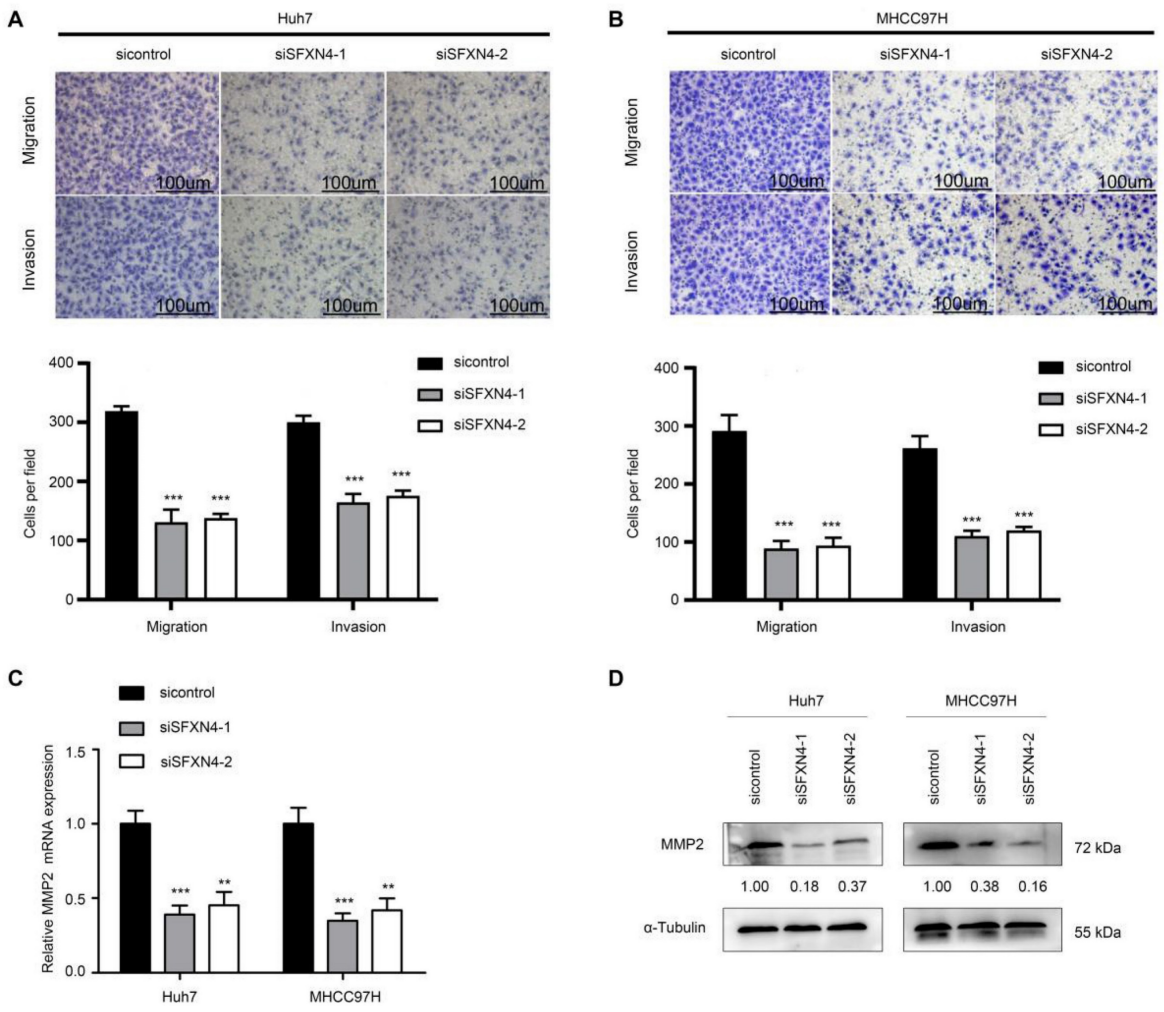
SFXN4 knockdown inhibits HCC migration and invasion. (A-B) Transwell assays were conducted to measure migration and invasion of Huh7 (A) and MHCC97H (B). (C-D) The qRT-PCR and Western blot were performed to detect MMP2 mRNA (C) and protein (D) expression in indicated cells. ^***^P<0.001, ^**^P<0.01. Scale bars: 100 μm.

**Figure 10:**
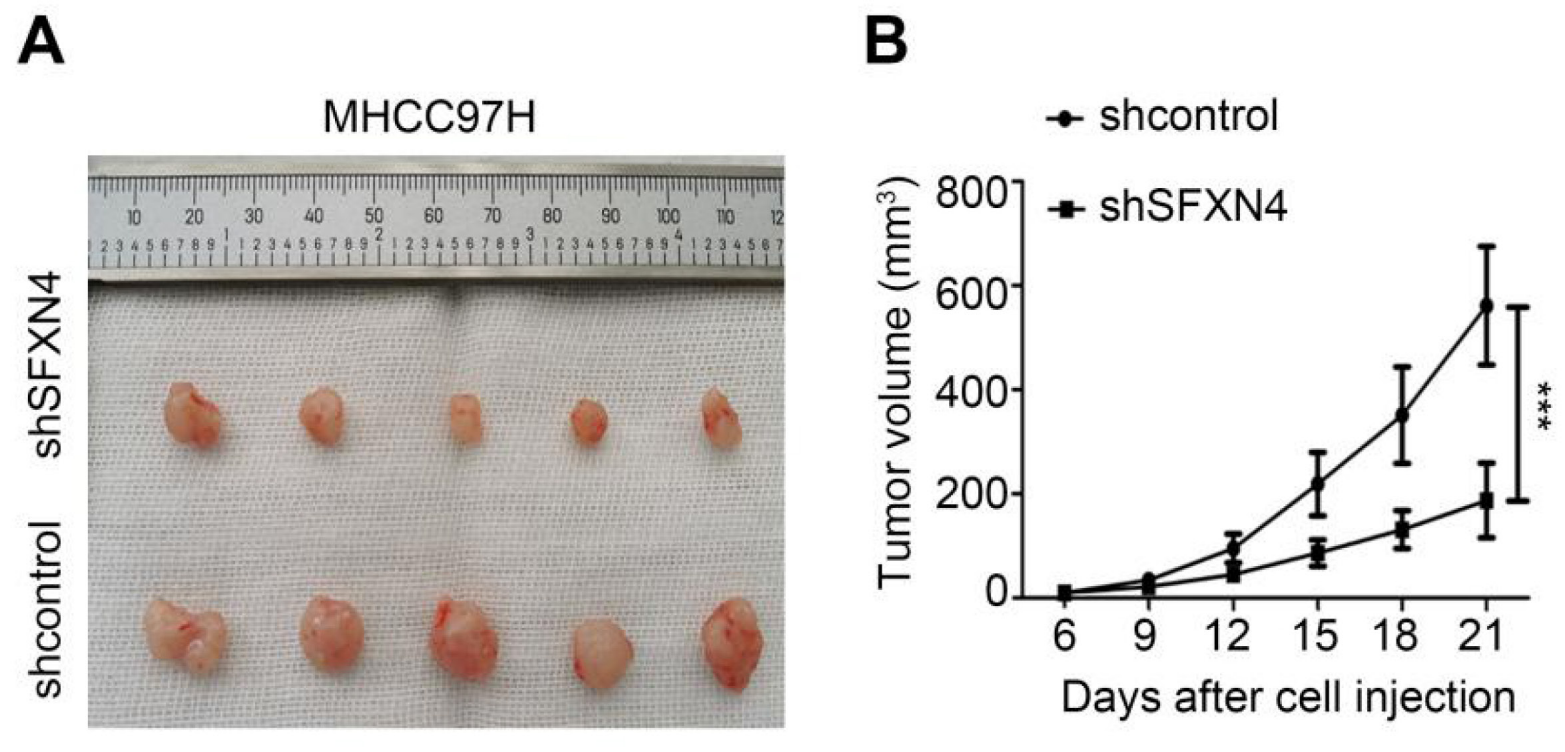
SFXN4 knockdown inhibits HCC growth *in vivo*. Appearance (A) and tumor growth curves (B) of subcutaneous tumors in mice. ^***^P<0.001.

To investigate the biological role of SFXN4 in HCC cell lines, we utilized two siRNA to knockdown SFXN4 in Huh7 and MHCC97H, and assessed the influence on cell proliferation, migration and invasion. By siRNA transfection, SFXN4 expression was dramatically knockdown (Figure 8A). To assess the role of SFXN4 on HCC proliferation, we performed CCK-8 analysis, which showed SFXN4 knockdown inhibited Huh7 and MHCC97H proliferation (Figure 8B), and these results were confirmed by EdU assay (Figure 8C). As a marker closely related to HCC proliferation, the mRNA and protein levels of cyclin D1 were decreased significantly upon knockdown of SFXN4 (Figure 8D-E). The results of Transwell assays showed SFXN4 knockdown restricted the migratory and invasive capability of Huh7 and MHCC97H (Figure 9A-B). Furthermore, SFXN4 inhibition dramatically decreased the expression of MMP2 (Figure 9C-D), which contributed to HCC migration and invasion. To verify the favorable role of SFXN4 in HCC proliferation, the mouse xenograft tumor assay were performed, by comparison of the appearance and tumor growth curves of subcutaneous tumors, we confirmed that knockdown of SFXN4 significantly hindered the growth of HCC (Figure 10A-B). Collectively, these data indicated that SFXN4 contributes to HCC growth and metastasis.

## Discussion

In the past 30 years, the risk of developing HCC has tripled in United States and other western countries, even worse, the risk is expected to increase due to the elevating incidence of non-alcoholic fatty liver disease and alcoholic hepatitis, while in Asian countries, HCC mainly develops in patients suffering viral infection, especially in those suffering chronic hepatitis B or hepatitis C 40. Despite great efforts in treatment, the prognosis of HCC patients is still dismal, thus making further elucidation of the mechanisms underlying HCC pathogenesis is in great urgency.

Recent studies highlighted that mitochondrial dysfunction affects the pathogenesis of in HCC 41, and mitochondrial-targeted drug delivery might be a promising therapeutic strategy 42. The SFXN proteins are a family of transporter inserted in the inner mitochondrial membrane 6. As the founding member, SFXN1 has proposed to be a mitochondrial transporter of serine 12, furthermore, SFXN1 mediated mitochondrial iron transportation and its overexpression led to mitochondrial dysfunction 8-11.

In our study, we found that, among the five SFXN members, SFXN4 expression was the highest and consistently elevated in HCC, and elevation of SFXN4 correlated significantly with HCC progression and prognosis. Generally, protein members of the same family have redundant function with each other. Similar to SFXN1, SFXN4 also regulated Fe-S cluster biogenesis, affected iron metabolism, and orchestrated mitochondrial respiration 22.

However, as the most divergent member, SFXN4 is different to SFXN1 and SFXN3, and it has no serine transport capacity 12. SFXN4 knockout in zebrafish caused mitochondrial respiratory defect and resulted in macrocytic anemia 23. Furthermore, mutation in SFXN4 is correlated with prenatal onset of mitochondrial diseases, and presented with macrocytic anemia and optic nerve hypoplasia 43, 44. Recent studies revealed that SFXN1 and SFXN4 regulate mitochondrial respiratory chain through different mechanisms, SFXN1 was reported to the maintain the integrity of mitochondrial complex Ⅲ 14, while SFXN4 coordinates the assembly of complex Ⅰ by interacting with MCIA complex, integrating mtDNA-encoded ND6 and promoting ND2-module maturation 45. These studies indicated that SFXN4 might has function distinct to other SFXN members 46. Indeed, in this study, we found SFXN4 expression has no significant correlation with other four member (Figure 5A), and the function of SFXN4 is related to oxidative phosphorylation, reactive oxygen species and metabolic pathways.

Although studies of SFXN members mainly focused on SFXN1, it is appealing that SFXN4 implicated in both tumor and non-tumor diseases. By trait correlated expression, SFXN4 was identified as a candidate casual gene regulating intramuscular fat 47. By GWAS meta-analysis, SFXN4 SNP (rs150807690) was found to be implicated in stroke, suggesting SFXN4 was a potential ischemic stroke loci11 48. Based on bioinformatics analysis, it was found that SFXN4 expression was upregulated during acute myocardial infarction 44. Recently, Dang et al found that SFXN4 was upregulated in lung adenocarcinoma, and it had significant correlation with immune infiltration 49. In our study, we also found that SFXN4 significantly correlated with multiple immune cells infiltration in HCC. Further, SFXN4 expression was elevated in ovarian cancer, and knockout of SFXN4 inhibited Fe-S biogenesis, decreased DNA repair, and inhibited tumor growth in ovarian cancer 50. Similarly, our study showed knockdown of SFXN4 inhibited HCC proliferation, migration and invasion, indicating that SFXN4 may be a promising target for therapeutic intervention.

To reveal the regulatory mechanism upstream and downstream of SFXN4, we explored the NetworkAnalyst and GSCALite database. We found that multiple transcription factors and miRNA affected the expression of SFXN4, and SFXN4 might exert its role by regulating various signaling pathways, such as EMT, PI3K/AKT and RAS/MAPK pathway. Furthermore, SFXN4 was predicted to be responsive or resistant to several small molecule inhibitors, such as TGX221, Vorinostat and Dasatinib. The drug sensitivity analysis of SFXN4 to these drugs was attractive; however, to confirm their effects, further studies are required.

In summary, SFXN4 is determined as an oncogene in HCC, SFXN4 may promote HCC development and progression via different mechanisms, indicating that SFXN4 may be a potential prognostic biomarker and therapeutic target in HCC.

## Conclusions

In conclusion, this study highlights that SFXN4 is overexpressed in HCC and is correlated with poor survival of HCC patients. SFXN4 is regulated by various transcription factors and miRNAs, and SFXN4 might exert its role through different mechanisms, including regulating oxidative phosphorylation, reactive oxygen species and metabolic pathways, immune infiltration, and cancer pathway activity. Knockdown of SFXN4 expression could inhibit proliferation, migration, and invasion of HCC *in vitro*, and inhibit HCC growth *in vivo*. Our study indicates SFXN4 may be a potential prognostic biomarker and therapeutic target in HCC.

## Supplementary Material

Supplementary figures.

## Figures and Tables

**Table 1:** Analyses of SFXN family expression in non-tumor (NT) and hepatocellular carcinoma (HCC) tissues from Gene Expression Omnibus (GEO) database. N: number, FC: fold change, P: P value.

		SFXN1		SFXN2		SFXN3		SFXN4		SFXN5	
Source	N	FC	P	FC	P	FC	P	FC	P	FC	P
GSE25097	NT: 243 HCC: 268	0.774	< 0.0001	0.789	< 0.0001	1.066	0.1657	1.152	< 0.0001	1.031	0.4579
GSE6764	NT: 30 HCC: 35	0.929	0.2821	1.313	0.0011	0.918	0.2468	1.6	< 0.0001	1.04	0.6234
GSE36376	NT: 193 HCC: 240	0.985	0.0041	0.979	0.0002	1.002	0.3474	1.023	< 0.0001	0.979	0.0061
GSE54236	NT: 80 HCC: 81	0.966	0.0002	0.969	0.0257	1.028	0.4243	1.017	0.013	0.963	< 0.0001
GSE63898	NT: 168 HCC: 228	0.983	0.0573	0.995	0.6312	1	0.9446	1.064	< 0.0001	0.996	0.6701
GSE76427	NT: 52 HCC: 115	0.925	0.115	0.764	< 0.0001	0.946	0.001	1.109	< 0.0001	0.598	< 0.0001

**Table 2:** Correlation between SFXN4 expression and clinicopathological characteristics of HCC patients in TCGA database.

Characteristic	Low expression of SFXN4	High expression of SFXN4	*P* value
n	187	187	
T stage, n (%)			0.666
T1	93 (25.1%)	90 (24.3%)	
T2	43 (11.6%)	52 (14%)	
T3	40 (10.8%)	40 (10.8%)	
T4	8 (2.2%)	5 (1.3%)	
N stage, n (%)			0.123
N0	124 (48.1%)	130 (50.4%)	
N1	0 (0%)	4 (1.6%)	
M stage, n (%)			1.000
M0	123 (45.2%)	145 (53.3%)	
M1	2 (0.7%)	2 (0.7%)	
Pathologic stage, n (%)			0.817
Stage I	89 (25.4%)	84 (24%)	
Stage II	40 (11.4%)	47 (13.4%)	
Stage III	41 (11.7%)	44 (12.6%)	
Stage IV	2 (0.6%)	3 (0.9%)	
Tumor status, n (%)			0.434
Tumor free	106 (29.9%)	96 (27%)	
With tumor	73 (20.6%)	80 (22.5%)	
Gender, n (%)			0.658
Female	63 (16.8%)	58 (15.5%)	
Male	124 (33.2%)	129 (34.5%)	
Race, n (%)			**0.004**
Asian	65 (18%)	95 (26.2%)	
Black or African American	11 (3%)	6 (1.7%)	
White	106 (29.3%)	79 (21.8%)	
Age, n (%)			0.380
<= 60	84 (22.5%)	93 (24.9%)	
> 60	103 (27.6%)	93 (24.9%)	
Weight, n (%)			**0.007**
<= 70	78 (22.5%)	106 (30.6%)	
> 70	93 (26.9%)	69 (19.9%)	
Height, n (%)			0.437
< 170	95 (27.9%)	106 (31.1%)	
>= 170	73 (21.4%)	67 (19.6%)	
BMI, n (%)			**0.008**
<= 25	75 (22.3%)	102 (30.3%)	
> 25	92 (27.3%)	68 (20.2%)	
Residual tumor, n (%)			1.000
R0	161 (46.7%)	166 (48.1%)	
R1	8 (2.3%)	9 (2.6%)	
R2	0 (0%)	1 (0.3%)	
Histologic grade, n (%)			**0.003**
G1	34 (9.2%)	21 (5.7%)	
G2	95 (25.7%)	83 (22.5%)	
G3	54 (14.6%)	70 (19%)	
G4	1 (0.3%)	11 (3%)	
Adjacent hepatic tissue inflammation, n (%)			0.883
None	65 (27.4%)	53 (22.4%)	
Mild	53 (22.4%)	48 (20.3%)	
Severe	9 (3.8%)	9 (3.8%)	
AFP (ng/ml), n (%)			**< 0.001**
<= 400	118 (42.1%)	97 (34.6%)	
> 400	19 (6.8%)	46 (16.4%)	
Albumin(g/dl), n (%)			0.460
< 3.5	32 (10.7%)	37 (12.3%)	
>= 3.5	121 (40.3%)	110 (36.7%)	
Prothrombin time, n (%)			0.470
<= 4	101 (34%)	107 (36%)	
> 4	48 (16.2%)	41 (13.8%)	
Child-Pugh grade, n (%)			0.809
A	99 (41.1%)	120 (49.8%)	
B	10 (4.1%)	11 (4.6%)	
C	1 (0.4%)	0 (0%)	
Fibrosis ishak score, n (%)			0.124
0	47 (21.9%)	28 (13%)	
1/2	14 (6.5%)	17 (7.9%)	
3/4	12 (5.6%)	16 (7.4%)	
5/6	38 (17.7%)	43 (20%)	
Vascular invasion, n (%)			0.075
No	114 (35.8%)	94 (29.6%)	
Yes	48 (15.1%)	62 (19.5%)	

**Table 3:** SFXN4 co-expression genes (TOP50) in Liver Hepatocellular Carcinoma (TCGA, PanCancer Atlas, 348 patients/samples)

Correlated gene	Cytoband	Spearman's Correlation	p-value	q-value
ATP5MK	10q24.33	0.750	3.57e-64	7.12e-60
MRPS16	10q22.2	0.670	1.30e-46	1.30e-42
BCCIP	10q26.2	0.664	1.10e-45	7.28e-42
EXOSC1	10q24.1	0.660	6.89e-45	3.43e-41
MRPL43	10q24.31	0.657	2.19e-44	8.74e-41
COX6B1	19q13.12	0.643	5.40e-42	1.80e-38
MTG1	10q26.3	0.639	2.18e-41	6.21e-38
NDUFB9	8q24.13	0.626	3.01e-39	7.50e-36
NDUFB8	10q24.31	0.621	1.68e-38	3.73e-35
VDAC2	10q22.2	0.620	2.47e-38	4.92e-35
SYNE2	14q23.2	-0.617	6.21e-38	1.13e-34
ATP5MF	7q22.1	0.617	8.12e-38	1.35e-34
MRPS24	7p13	0.613	2.75e-37	4.22e-34
UQCRB	8q22.1	0.613	2.98e-37	4.25e-34
RPS24	10q22.3	0.608	1.73e-36	2.30e-33
EFCAB14	1p33	-0.606	3.26e-36	4.06e-33
NDUFA3	19q13.42	0.604	5.94e-36	6.90e-33
HIGD2A	5q35.2	0.604	6.22e-36	6.90e-33
BMPR2	2q33.1-q33.2	-0.603	9.00e-36	9.44e-33
PPP1R35	7q22.1	0.602	1.15e-35	1.15e-32
ATP5F1E	20q13.32	0.597	5.49e-35	5.21e-32
FRMD4B	3p14.1	-0.597	5.76e-35	5.22e-32
NDUFA13	19p13.11	0.595	9.92e-35	8.60e-32
EXOC5	14q22.3	-0.595	1.22e-34	1.01e-31
ATP5ME	4p16.3	0.594	1.29e-34	1.03e-31
MRPS17	7p11.2	0.594	1.52e-34	1.16e-31
EIF3K	19q13.2	0.592	2.44e-34	1.80e-31
GPATCH2L	14q24.3	-0.592	2.71e-34	1.93e-31
COX6C	8q22.2	0.591	4.33e-34	2.98e-31
LBHD1	11q12.3	0.590	5.17e-34	3.40e-31
PPIA	7p13	0.590	5.29e-34	3.40e-31
HSPE1	2q33.1	0.589	6.70e-34	4.06e-31
MRPL22	5q33.2	0.589	6.71e-34	4.06e-31
USP12	13q12.13	-0.588	9.24e-34	5.42e-31
ZXDA	Xp11.21	-0.587	1.19e-33	6.78e-31
MRPS12	19q13.2	0.586	1.67e-33	9.26e-31
MAP3K2	2q14.3	-0.586	1.77e-33	9.52e-31
RNF168	3q29	-0.585	2.60e-33	1.36e-30
SOS2	14q21.3	-0.584	3.14e-33	1.61e-30
SMARCA2	9p24.3	-0.584	3.50e-33	1.75e-30
PIK3CA	3q26.32	-0.583	4.62e-33	2.23e-30
UQCRBP1	Xp11.21	0.583	4.71e-33	2.23e-30
GEMIN7	19q13.32	0.583	5.17e-33	2.40e-30
COA6	1q42.2	0.581	9.01e-33	4.09e-30
POLR2H	3q27.1	0.577	2.48e-32	1.10e-29
DDI2	1p36.21	-0.574	6.95e-32	3.02e-29
CCNT1	12q13.11-q13.12	-0.574	7.91e-32	3.36e-29
ROMO1	20q11.22	0.573	8.51e-32	3.53e-29
NDUFS3	11p11.2	0.572	1.09e-31	4.42e-29
CREBBP	16p13.3	-0.572	1.23e-31	4.89e-29

## Abbreviations

HCC: Hepatocellular carcinoma
SFXN: Sideroflexin
SLC56: Solute carrier 56
Fe^2+^: Iron
GTEx: Genotypic Tissue Expression
TCGA: The Cancer Genome Atlas
GEPIA: Gene Expression Profiling Interactive Analysis
GEO: Gene expression omnibus
GO: Gene Ontology
KEGG: Kyoto Encyclopedia of Genes and Genome
ssGSEA: single-sample gene set enrichment analysis
CCK-8: Cell Counting Kit-8
EdU: 5-ethynyl-2'-deoxyuridine
MMP2: matrix metalloproteinase 2
