# Supplementary material for: Comprehensive Analysis of Sideroflexin 4 in Hepatocellular Carcinoma by Bioinformatics and Experiments
Source: Int J Med Sci. 2023 Aug 21;20(10):1300–15. doi: 10.7150/ijms.86990 (PMC10542026; doi:10.7150/ijms.86990)
Supplement: Supplementary file 1 — Supplementary figures. [file ijmsv20p1300s1.pdf]

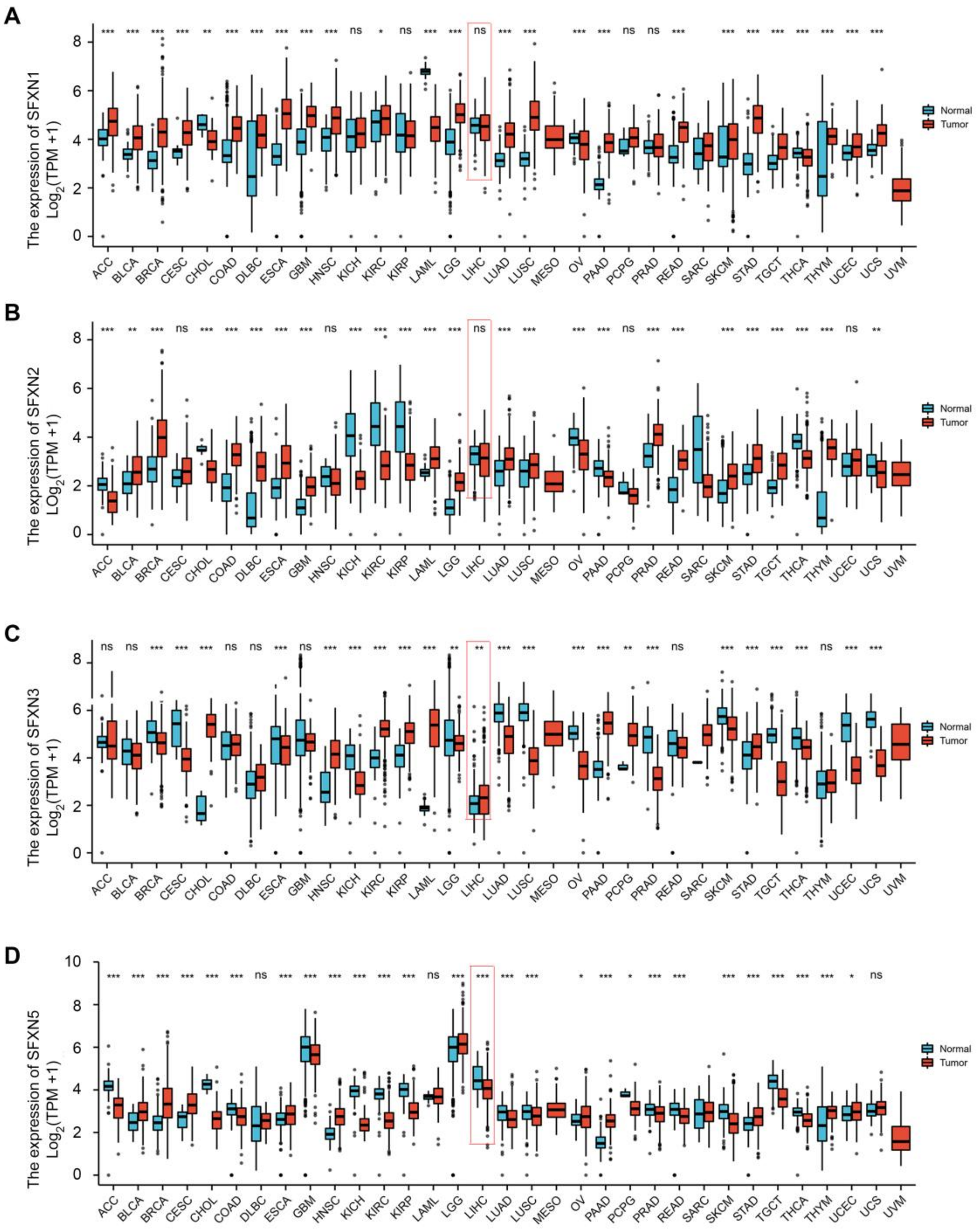

Figure S1. Expression of SFXN members in pan-cancer from XENA-TCGA_GTEx database. (A) SFXN1. (B) SFXN2. (C) SFXN3. (D) SFXN5. $^{*}P<0.05$, $^{**}P<0.01$, $^{***}P<0.001$, $^{ns}P>0.05$.

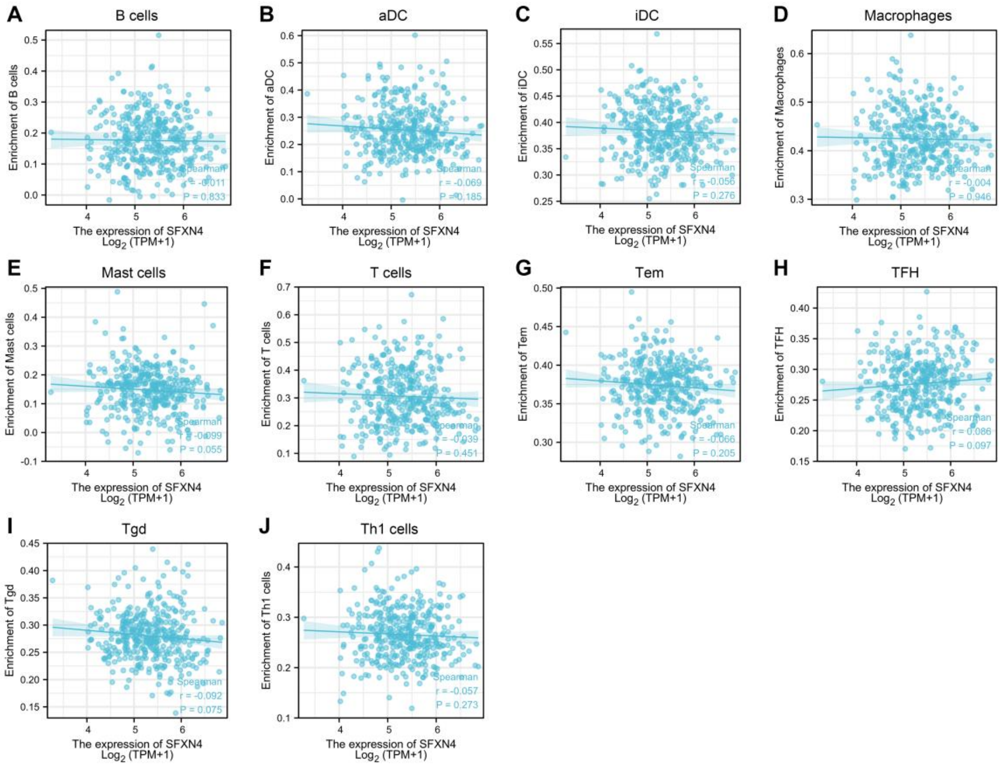

Figure S2. Correlation of SFXN4 expression with immune infiltration in HCC. (A) B cells. (B) aDC. (C) iDC. (D) Macrophages. (E) Mast cells. (F). T cells. (G) Tem. (H) TFH. (I) Tgd. (J) Th1 cells. The data were acquired from ssGSEA, and $P > 0.05$ was considered statistically not significant.
